# Severe skin and soft tissue infection in the left upper limb caused by *Aeromonas veronii*: a case report

**DOI:** 10.1186/s13256-023-03770-y

**Published:** 2023-01-30

**Authors:** Linhui Li, Jie Huang, Long Xu, Guangyi Wang, Shichu Xiao, Zhaofan Xia, Qin Qin, Yazhou Li, Shizhao Ji

**Affiliations:** 1 Department of Burns, Burn Institute of PLA, The First Affiliated Hospital of Naval Medical University, No. 168 Changhai Road, Yangpu District, Shanghai, 200433 China; 2 Department of Laboratory Diagnosis, The First Affiliated Hospital of Naval Medical University, No. 168 Changhai Road, Yangpu District, Shanghai, 200433 China

**Keywords:** *Aeromonas veronii*, Wound infection, Antibiotic therapy, Whole-genome sequencing, Case report

## Abstract

**Introduction:**

Skin and soft tissue infections are common because of exposure to aquatic environment, while severe infections caused by *Aeromonas veronii* are rare.

**Case presentation:**

We report a case of severe skin and soft tissue infection of the left upper limb caused by *Aeromonas veronii*. A 50-year-old Chinese woman, who had a history of cardiac disease and type 2 diabetes mellitus, accidentally injured her left thumb while cutting a fish. Early antibiotic therapy and surgical debridement was performed before the result of bacterial culture came back. Whole-genome sequencing was further performed to confirm the pathogen and reveal the drug resistance and virulence genes. The wound was gradually repaired after 1 month of treatment, and the left hand recovered well in appearance and function after 3 months of rehabilitation.

**Conclusion:**

Early diagnosis, surgical intervention, and administration of appropriate antibiotics are crucial for patients who are suspected of having skin and soft tissue infection, or septicemia caused by *Aeromonas veronii*.

## Introduction

*Aeromonas* is a genus of Gram-negative bacilli widely distributed in aquatic environments and can infect the human body through the fecal–oral route or wounds to cause diseases, mainly manifesting as gastroenteritis, wound infections, and septicemia [1]. We report a case of severe infection by *Aeromonas veronii* in the left upper limb caused by accidental injury to the left thumb while cutting a fish.

## Case presentation

A 50-year-old Chinese woman accidentally injured her left thumb at 5 pm, while cutting a fish for dinner. She washed the wound with cold, running water for 1 minute and applied a bandage. However, on the night of the incident, her left thumb became red and swollen, which gradually spread to the left dorsal hand and extended to the forearm within 12 hours after the injury, warranting a visit to the emergency department of local hospital the next morning. The doctor recommended hospitalization for surgery. She refused and came to our hospital for treatment a day after the injury.

**Fig. 1:**
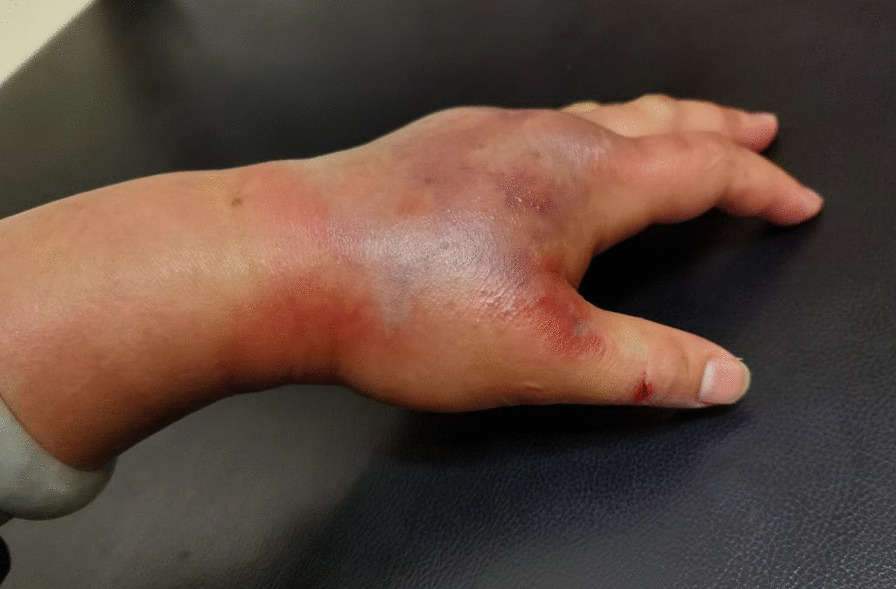
A linear wound observed on the left thumb and the left hand showed redness, swelling, purple ecchymosis, and tiny tension blisters

During admission, there was significant swelling on the dorsum of the left hand and left forearm, high tension, and severe infection. She had a history of dilated cardiomyopathy, chronic heart failure, atrial fibrillation, and type 2 diabetes. Physical examination on admission showed a temperature of 36.8 °C, pulse rate of 80 beats per minute, respiratory rate of 20 breaths per minute, and blood pressure of 87/54 mmHg. A linear wound was also observed at approximately 0.5 cm in length at the left thumb interphalangeal joint on the radial side. The skin and soft tissues of the left hand and the left forearm were red and swollen, especially the left dorsal hand extending to the left dorsal forearm. A purple ecchymosis was noted on the skin of the left dorsal hand, accompanied by scattered tiny tension blisters, high skin tension, significant tenderness, slightly above normal skin temperature, and a weak pulse in the ulnar and radial arteries (Fig. 1). Emergency laboratory and clinical examinations performed revealed white blood cell count (WBC) of 21.41 × 10^9^/L, N of 92.0%, platelet count (PCT) of 6.72 ng/mL, GLU of 16.4 mmol/L, HbA1c of 8.0%, and BNP > 5002 pg/mL. A standard 12-lead electrocardiogram (ECG) showed atrial fibrillation, while a plain computerized tomography (CT) scan of the left forearm and left hand revealed moderate to severe swollen skin and soft tissue.

Initial diagnosis was skin and soft tissue infection in the left upper limb, dilated cardiomyopathy, congestive heart failure (CHF) (grade III cardiac function), atrial fibrillation, postoperative state of cardiac pacemaker implantation, and type 2 diabetes mellitus (DM). Combining the patient’s history of injury, the possibility of infection by aquatic-related bacteria such as *Vibrio*, *Aeromonas*, *Mycobacterium marinum* was considered [2].

**Fig. 2:**
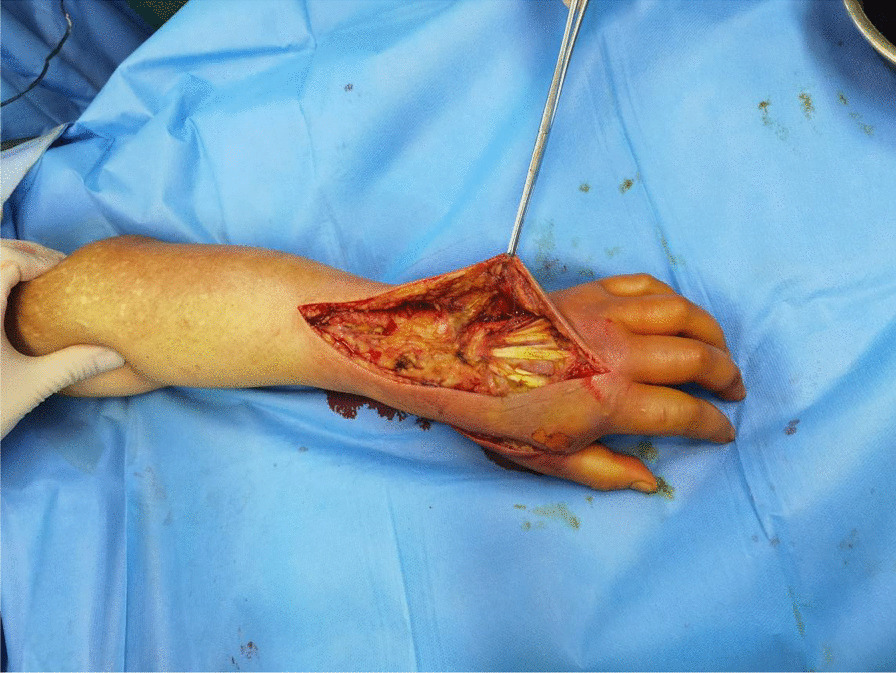
A longitudinal incision was made to achieve complete tension reduction, and the discharge was sent for bacterial culture

**Fig. 3:**
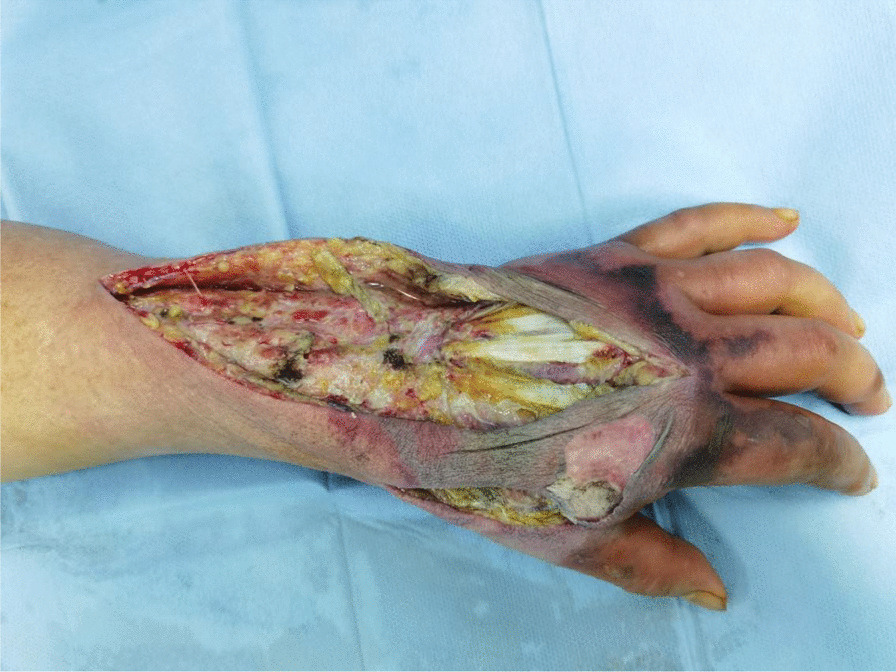
Wound infection continued to spread to the fingers, accompanied by skin and soft tissue necrosis

**Fig. 4:**
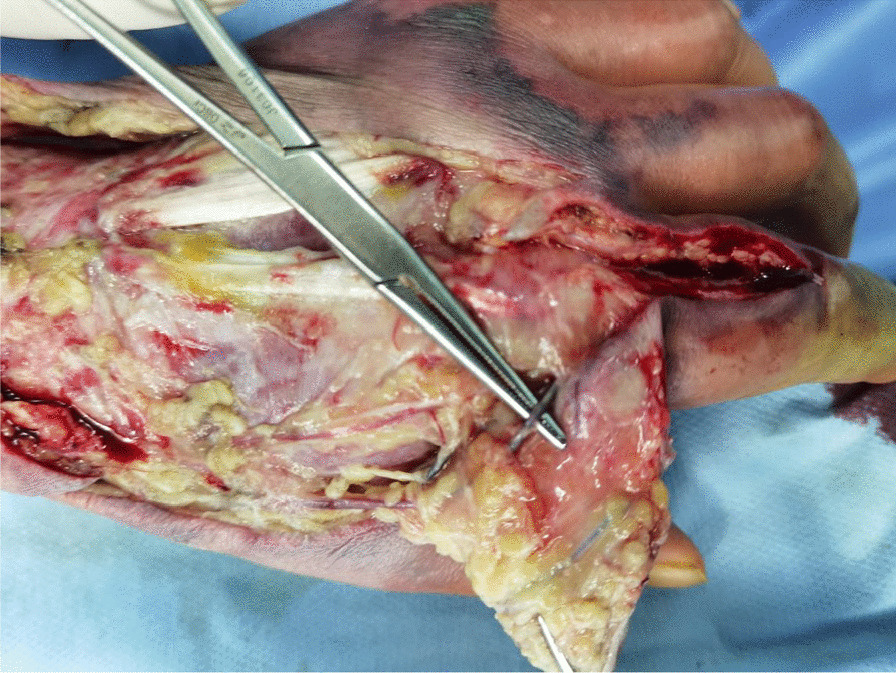
Subcutaneous venous network was embolized

**Fig. 5:**
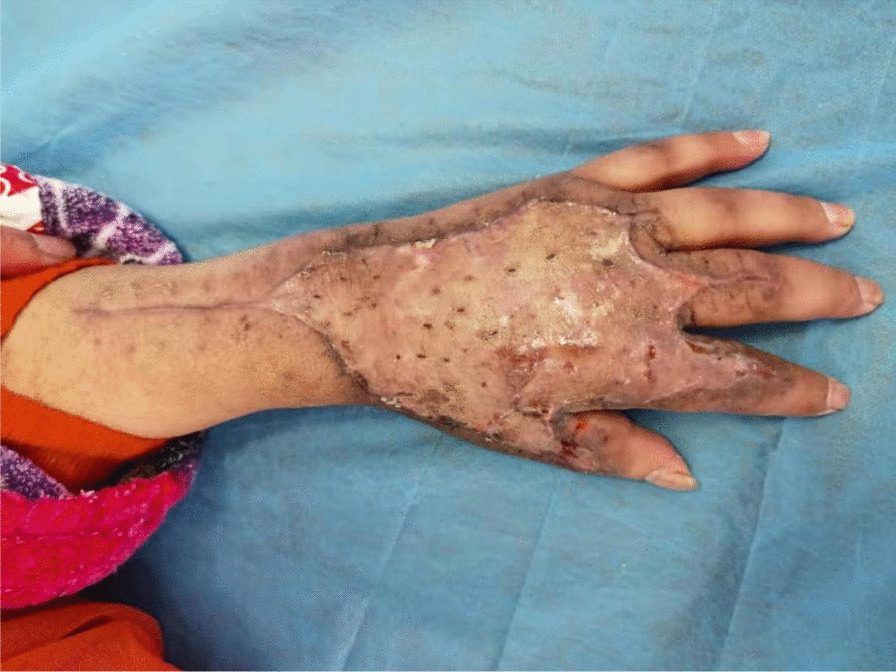
After 3 months of follow-up, the left hand recovered well in appearance and function

Emergency open decompression was performed on the left upper limb. A longitudinal incision was made to the deep fascial layer, along the most significantly swollen site, to achieve complete tension reduction and drainage (Fig. 2). Intraoperatively, a large amount of clear yellow discharge was found in the deep fascia of the dorsal area of the left wrist and hand. We sent the discharge for bacterial culture. The wound was thoroughly flushed with a large amount of hydrogen peroxide, normal saline, and iodophor and then packed and bandaged with iodoform gauze. Postoperatively, we administered antibiotics such as cefoperazone–sulbactam sodium 3 g intravenously twice a day, combined with moxifloxacin 0.4 g once a day. During postoperative change of dressing, we found that the wound infection continued to spread, partly to the second phalanxes, accompanied by skin blackening and tissue necrosis (Fig. 3). On the second day after admission, surgical debridement was performed on the left upper limb. Further open decompression and drainage was conducted. During the operation, the viability of fat and tendon tissues was found to be poor and the subcutaneous venous network was scattered and embolized (Fig. 4); therefore, another discharge sample was sent for bacterial culture. The wound was flushed with a large amount of hydrogen peroxide, normal saline, and iodine and then packed and bandaged with iodoform gauze. On the fourth day after admission, the results of bacterial culture were reported as *A. veronii* and *A. ichthiosmia*. The drug sensitivity test results showed that the two bacteria were sensitive to cephalosporins, aminoglycosides, and quinolones but resistant to penicillin and carbapenems. Based on the drug sensitivity test results of the bacterial culture, we administered cefoperazone–sulbactam sodium 3 g intravenously twice daily (BID), combined with levofloxacin 0.5 g four times daily (QD). Subsequently, multiple operations to control the wound infection, and further skin grafting to close the wound, were performed. Finally, the patient recovered and was discharged 1 month after the injury. After 3 months of rehabilitation, the left hand recovered well in appearance and function (Fig. 5). The patient was very satisfied with our treatment and expressed great gratitude.

## Discussion

To further confirm whether the infection was caused by the pathogenic bacteria carried by the fish, we tried to track the original fish, but it was no longer available. We then caught the same species of fish from the same river for bacterial culture. We found that the main parasitic bacteria on the fish surface was *A. veronii*, which was consistent with the bacterial culture results of the patient. Therefore, it was preliminarily determined that the infectious bacteria in this patient came from the fish. However, the drug sensitivity test results showed that *A. veronii* was sensitive to all drugs, including penicillin and carbapenem antibiotics, which was inconsistent with the drug sensitivity test results of the cultured bacteria from the wound of our patient. We then conducted whole-genome sequencing of the cultured *A. veronii* and *A. ichthiosmia* from the wound surface, and cultured *A. veronii* from the fish skin surface to clarify the difference in drug resistance between these two bacteria. The results showed that both cultured bacteria from the wound surface were *A. veronii*. *A. veronii* and *A. ichthiosmia* are highly homologous in gene sequences, so it is sometimes difficult to distinguish them by general bacterial culture, or by using 16S rRNA [3]. And there are 27 drug-resistant genes in the *A. veronii*, including the CphA and OXA genes (those that produce metallo-β-lactamase and penicillinase, which can produce drug resistance by hydrolyzing carbapenem and penicillin antibiotics) [4, 5]. However, no drug-resistant genes were found from the cultured *A. veronii* from the fish skin surface.

In recent years, with the wide application of antibiotics in clinical practice and aquaculture, several studies have reported that *Aeromonas* has evolved and mutated to contain drug-resistant genes, and that *A. veronii* showed high drug resistance to penicillin (except piperacillin) and carbapenem antibiotics [6, 7]. Sanchez-Cespedes* et al*. reported a case of cholangitis caused by *A. veronii*, in which the drug sensitivity test results showed resistance to imipenem, considered to be caused by the patient’s long-term use of penicillin antibiotics. This induced carbapenem resistance of the *A. veronii* in the human bile duct [8]. In our case, the patient was not on antibiotics before admission and when the first bacterial culture was taken, with the time from the injury to the first bacterial culture was < 36 h; therefore, we did not consider drug resistance caused by a bacterial gene mutation in the human body. Further investigation revealed that sulfonamides and macrolides had been used in the aquaculture lake for a long time, which is the possible reason for *A. veronii*’s resistance to penicillin and carbapenem antibiotics.

To date, several studies have reported on human infections caused by *A. veronii*, mainly in patients with immune disorders such as acute and chronic lymphoblastic leukemia, acute myeloid leukemia, acquired immunodeficiency syndrome, and DM. This often results in severe septicemia and mortality [9]. Our patient had a history of underlying diseases, such as DM and chronic heart disease, which might be an important factor causing the susceptibility and serious illness after infection. In addition, the whole-genome sequencing showed that the cultured *A. veronii* from the fish skin contained only the virulence genes *act*, *aerA*, *hlyA*, and *fla*, whereas that from the wound surface contained the virulence genes *laf*, *lip*, *ascV*, *aexT*, and *ascF-ascG* in addition to the aforementioned virulence genes. These genes promote bacterial invasion and release of virulence factors, and induce a nonspecific inflammatory response in the human body. This may be the reason *A. veronii* has strong virulence and pathogenicity, which leads to the rapid progress of patients’ illnesses [10, 11].

The spectrum of *Aeromonas* infections mainly includes gastroenteritis, wound infections, and septicemia. *A. veronii* has also been reported to generate disorders in several other organs, such as hepatobiliary organs, lungs, kidneys, and bones [12–15]. With regards to antibiotic management, *Aeromonas* is susceptible to monobactams, carbapenems, third- and fourth-generation cephalosporins, aminoglycosides, and fluoroquinolones [16]. Given that there is an increase in resistance to beta-lactam antibiotics owing to the presence of genes, and that *A. veronii* is commonly beta-lactamase producing, treatment with broad-spectrum beta-lactams is not recommended [17]. So third- and fourth-generation cephalosporins and fluoroquinolones should be considered first for the treatment of infections caused by *A. veronii* [18]. Gram stain, culture results, and gene sequencing of the exudate may provide information to guide antibiotic therapy.

In summary, the patient with cardiac disease and type 2 diabetes succeeded in defeating *A. veronii* with the help of decisive surgery and proper drugs. Gene sequencing, to some extent, can compensate for the shortcomings of bacterial culture, and assist in the diagnosis and treatment of severe skin and soft tissue infection on a genetic level.

## Conclusions

We should be alert to possible *Aeromonas* infection in patients suspected of having skin and soft tissue infection or septicemia caused by aquatic-related bacteria. Moreover, early diagnosis, surgical intervention, and administration of appropriate antibiotics are crucial for patients’ good prognosis.

## Data Availability

All list authors agreed data sharing to this article.
